# The role of endothelial TRP channels in age-related vascular cognitive impairment and dementia

**DOI:** 10.3389/fnagi.2023.1149820

**Published:** 2023-03-20

**Authors:** Sharon Negri, Madison Sanford, Helen Shi, Stefano Tarantini

**Affiliations:** ^1^Vascular Cognitive Impairment and Neurodegeneration Program, Reynolds Oklahoma Center on Aging/Center for Geroscience and Healthy Brain Aging, Department of Neurosurgery, University of Oklahoma Health Sciences Center, Oklahoma City, OK, United States; ^2^Stephenson Cancer Center, University of Oklahoma Health Sciences Center, Oklahoma City, OK, United States; ^3^International Training Program in Geroscience, Doctoral School of Basic and Translational Medicine/Department of Public Health, Semmelweis University, Budapest, Hungary; ^4^Department of Health Promotion Sciences, College of Public Health, University of Oklahoma Health Sciences Center, Oklahoma City, OK, United States

**Keywords:** aging, neurodegeneration, neurovascular coupling, Geroscience, cognitive dysfunction, dementia, lifestyle intervention

## Abstract

Transient receptor potential (TRP) proteins are part of a superfamily of polymodal cation channels that can be activated by mechanical, physical, and chemical stimuli. In the vascular endothelium, TRP channels regulate two fundamental parameters: the membrane potential and the intracellular Ca^2+^ concentration [(Ca^2+^)_i_]. TRP channels are widely expressed in the cerebrovascular endothelium, and are emerging as important mediators of several brain microvascular functions (e.g., neurovascular coupling, endothelial function, and blood–brain barrier permeability), which become impaired with aging. Aging is the most significant risk factor for vascular cognitive impairment (VCI), and the number of individuals affected by VCI is expected to exponentially increase in the coming decades. Yet, there are currently no preventative or therapeutic treatments available against the development and progression of VCI. In this review, we discuss the involvement of endothelial TRP channels in diverse physiological processes in the brain as well as in the pathogenesis of age-related VCI to explore future potential neuroprotective strategies.

## 1. Introduction

Age-related cognitive decline is a growing world health problem, which preferentially affects elderly people greater than the age of 60 years old. This unprecedented increase is caused by the continuous increase of life expectancy, and the consequent survival of a growing number of elderly individuals worldwide. Cognitive decline impairs memory, comprehension, learning capacity, and judgment, and has been demonstrated to be closely associated with several types of cerebrovascular pathologies, such as stroke and atherosclerosis. According to the World Health Organization (WHO), dementia is the seventh leading cause of death worldwide. It affected 55 million people in 2021, and is expected to reach 78 million in 2030 and 139 million in 2050 (World Health Organization, 2022). Notably, Alzheimer’s disease (AD) and vascular dementia (VaD) are, respectively, the first and second most common forms of dementia (Iadecola, 2013). In 1993, the term vascular cognitive impairment (VCI) was introduced to encompass any type of cognitive disease associated with cerebrovascular disorders, without considering the underlying mechanism; this terminology helped to emphasize the increasingly understood contribution of cerebrovascular function to cognitive health (Dichgans and Leys, 2017; Balasubramanian et al., 2020). Since then, it has been well accepted that cerebral microvascular dysfunction characterizes cognitive decline in neurodegenerative disorders, particularly in AD and VaD (Dichgans and Leys, 2017; Balasubramanian et al., 2020).

Brain endothelial cells (BECs) have lately emerged as important players in age-related neurogenesis, neuroinflammation and cognitive function (Chen et al., 2020). For instance, several chemical [e.g., neurotransmitters and reactive oxygen species (ROS)] and mechanical (e.g., shear stress) stimuli induce intracellular Ca^2+^ signals in BECs (Guerra et al., 2018; Zuccolo et al., 2018; Berra-Romani et al., 2019a,b; Negri et al., 2019). The subsequent increase in intracellular Ca^2+^ concentration [(Ca^2+^)_i_] leads to the production of multiple Ca^2+^-dependent vasoactive mediators, such as nitric oxide (NO), prostacyclin, prostaglandin H2, and thromboxane A2 (Guerra et al., 2018). Moreover, the increase in [Ca^2+^]_i_ may activate intermediate and small-conductance Ca^2+^-dependent K^+^ channels (IK and SK, respectively), by inducing a membrane hyperpolarization that is conveyed to vascular smooth muscle cells through myoendothelial junctions (MEGJs), causing vasodilatation (Behringer, 2017; Garland and Dora, 2017). Notably, BECs, especially capillary BECs, present wide transcriptional and functional alterations in normal aging, by increasing innate immunity and oxidative stress response pathways. Moreover, it is well known that BECs’ Ca^2+^ machinery undergoes major changes following cerebrovascular damage in several cardiovascular disorders (e.g., AD and VaD; Iadecola, 2004, 2013; Cascella and Cecchi, 2021). Finally, by being the key constituent elements of the blood brain barrier (BBB), BECs are exposed to a wide range of circulatory proteins, mechanical stimuli, and chemical molecules from different compartments of the organism (Chen et al., 2020).

In this view, organisms have developed several strategies to sense environmental changes: one of which is represented by the superfamily of the transient receptor potential (TRP) channels. They are polymodal cation channels that are activated by mechanical, physical, and chemical stimuli, and regulate two fundamental endothelial parameters: the membrane potential and the [Ca^2+^]_i_ (Samanta et al., 2018). The first TRP channel was discovered in a blind mutant of *Drosophila* in 1969 (Cosens and Manning, 1969), where the deletion of the *trp* gene caused impairment in the fly’s visual capacity and an alteration in its electrical response to light. Indeed, prolonged light exposure induced only a transient retinal depolarization in mutant drosophila photoreceptors, far from the normal steady-state depolarization recorded in wild-type flies (Earley and Brayden, 2015; Moran, 2018). The identity of the *trp* gene was unearthed several years later by Craig Montell and Gerald Rubin (Montell et al., 1985; Montell and Rubin, 1989). Six years later, three independent groups found TRP channel expression in vertebrates, and in particular, in mouse brains, xenopus oocytes (Petersen et al., 1995), and humans (Wes et al., 1995; Zhu et al., 1995). The first cloned mammalian TRP channel was denominated TRP Canonical 1 (TRPC1), due to its resemblance to the *Drosophila* TRP channel (Wes et al., 1995). Not surprisingly, the discovery of human TRPC1 started a 10 year long scientific race to identify other TRP channels. Nowadays, 28 members have been discovered, and are continuously studied in physiological and pathophysiological conditions as a promising therapeutic target for several neurological and cardiovascular disorders. TRP channels have emerged as important regulators of neurovascular functions, and their proper function is fundamental to maintain normal brain activity (Kuppusamy et al., 2021). In the last few decades, growing evidence highlights the importance of healthy lifestyle habits to prevent cardiovascular and neurovascular disorders typical of aging (Csipo et al., 2019; Balasubramanian et al., 2020). In this context, TRP channels have been tightly linked to some risk factors (e.g., obesity and diabetes) involved in the onset of these disorders (Moraes et al., 2021). It has been demonstrated that hyperglycemia and diabetes cause impaired endothelial-dependent vasodilatation(Makimattila et al., 1996) and endothelial dysfunction(Xu et al., 2003; Ren et al., 2017), which correlates with modifications in the expression and function of several TRP channels (Moraes et al., 2021). Moreover, it has long been known that the clearance of high levels of glucose and lipids in older adults is robustly decreased (Donato et al., 2015). Pathological increases in glucose and lipids cause oxidative stress and neuroinflammation, thereby leading to endothelial dysfunction typical of aging brains (Donato et al., 2015). This brings us to the last important issue: the role of dietary regimens (e.g., time restricted feeding and caloric restriction) in the prevention of VCI and cognitive decline (Csiszar et al., 2014; Balasubramanian et al., 2020). An appropriate fasting time may indeed resolve the acute metabolic insults (Donato et al., 2015), and similarly, specific food-derived compounds may ameliorate the aging-dependent vascular defects by targeting different TRP channels (Zhang et al., 2019). In this review, our focus will be on exploring the role of TRP channels in cerebrovascular mechanisms from various perspectives, with the aim of elucidating how targeted interventions could potentially enhance brain health and improve overall quality of life. Unlike traditional aging research which focused primarily on extending lifespan, modern aging research places a greater emphasis on improving the quality of life for individuals as they age.

## 2. TRP channels

### 2.1. Brief introduction

The TRP channel superfamily comprises 28 members subdivided in six subfamilies based on their sequence homology: TRP Canonical (TRPC1-7), TRP Mucolipin (TRPML1-3), TRP Ankyrin (TRPA1), TRP Melastatin (TRPM1-8), TRP Vanilloid (TRPV1-6), and TRP Polycystin (TRPP2, TRPP3, and TRPP5; Gees et al., 2010; Earley and Brayden, 2015; Smani et al., 2018). The TRP subunits consists of 553 to 2022 amino acids polypeptides (⁓ 64–230 kDa) organized in six transmembrane (TM1-6) α-helices with cytosolic NH_2_- and COOH-termini and a re-entrant loop between TM5 and TM6 (Negri et al., 2019). In particular, NH_2_- and COOH-extremities demonstrate very high variability in length and functions between the different subfamilies (Gaudet, 2008). For example: (i) TRPA, TRP, and TRPV NH_2_-terminals present a variable number of ankyrin repeats, which is important in sensing and gating (Gaudet, 2008); (ii) TRPC, TRPM, and TRPV present a 25-amino acid conserved element named TRP domain located distal to S6 and characterized by two highly conserved TRP-boxes, which delimit a more variable central region (Earley and Brayden, 2015); (iii) TRPM6 and TRPM7 COOH-terminals show an α-kinase domain that modify channel function (Zhang et al., 2014); (iv) TRPM2 COOH-extremity features an ADPR hydrolase domain (Nudix-like domain) (Scharenberg, 2005). Finally, (v) TRP channels may interact with multiple intracellular kinases and Ca^2+^-dependent sensors [i.e., Calmodulin (CaM) and Stromal Interacting Molecule 1 (STIM1)] due to the presence of CaM-inositol 1,4,5-trisphosphate receptors (InsP_3_R)-binding (CIRB) sites and Ca^2+^-binding EF hands domains at the COOH-extremity (Gaudet, 2008; Gees et al., 2010). Overall, the TRP channel’s structure is very similar to that of voltage-gated K^+^ channels, but TRP channels lack the series of charged amino acids in TM4, which explains their lower sensitivity to voltage changes. Moreover, the activation voltage-range is maintained at a non-physiological interval that is not usually experienced by the cell (Zheng, 2013). Notably, the TRP channel’s voltage response may be affected by other chemical and physical stimuli (e.g., pH variation, temperature, and agonists), which reconducts the voltage sensitivity to a physiological range (Voets et al., 2004). For instance, TRPV1 half-activation potential is +100 mV at room temperature; conversely, at 42°C, it decays to −50 mV, which is reachable by a sensory neuron (Voets et al., 2004). An analogous mechanism has also been characterized in TRPM8 (Voets et al., 2007) and TRPM4 and TRPM5 (Hofmann et al., 2003; Talavera et al., 2005).

The functional channel is composed of four subunits, which may aggregate into homomeric or heteromeric structures around a central pore delimitated by TM5, TM6, and the connecting re-entrant loop (Gaudet, 2008). TRP channels preferentially assemble into homomeric complexes; however, recent evidence demonstrates the presence of a large number of heteromeric channels in naive cells, including vascular endothelial cells (ECs; Zheng, 2013). Notably, heteromeric channels may be constituted by subunits from the same subfamily [e.g., TRPC1 aggregates with TRPC3, TRPC4 or TRPC5 (Smani et al., 2018)] or from different subfamilies [e.g., TRPC1/TRPP2 (Berrout et al., 2012) or TRPC1/TRPV4 complexes (Ma et al., 2011)].

### 2.2. Gating mechanisms and biophysical properties

Transient receptor potential channels are polymodal cation receptors that may be activated by multiple chemical and physical stimuli, including temperature variation, mechanical perturbation (e.g., laminar shear stress, and membrane stretch), exogenous dietary agonists (e.g., menthol, allyl isothiocyanate and capsaicin), hydrogen peroxide (H_2_O_2_), arachidonic acid (AA), intracellular ions (e.g., H^+^ and Ca^2+^), and finally, some TRP channels may be activated by the depletion of the endoplasmic reticulum (ER) Ca^2+^ store through interaction with STIM1 and Orai1, which are the main proteins involved in the activation of store-operated Ca^2+^ entry (SOCE)(Venkatachalam and Montell, 2007; Earley and Brayden, 2015; Ambudkar et al., 2017; Negri et al., 2019; Thakore and Earley, 2019). SOCE is mediated mainly by the Ca^2+^ release-activated Ca^2+^ (CRAC) channels, which comprises Orai1, and by the less selective store-operated Ca^2+^ (SOC) channels. After a long debate, TRPC1 is now considered a component of the SOC channels since it is involved in assembling the ternary complex with Orai1 and STIM1 (Jardin et al., 2008; Ambudkar et al., 2017).

Usually, TRP channels are defined as non-selective cation channels by driving both monovalent (i.e., Na^+^ and K^+^) and divalent (i.e., Ca^2+^ and Mg^2+^) cation fluxes, but their relative selectivity (P_Ca_/P_Na_) ranges from TRPM4 and TRPM5, which are substantially impermeable to Ca^2+^ (P_Ca_/P_Na_ < 0.01), to TRPV5 and TRPV6, which show higher Ca^2+^ permeability (P_Ca_/P_Na_ > 100) (Smani et al., 2018). The remaining fraction of TRP channels feature an intermediate selectivity, and some of them present a peculiar permeability to Mg^2+^ (e.g., TRPM6 and TRPM7), H^+^ (e.g., TRPV1, TRPML1 and TRPP3), and metal ions (e.g., manganese, zinc, barium, strontium nickel and cobalt; Gees et al., 2010; Smani et al., 2018).

## 3. Age-related cerebrovascular changes

Aging has been demonstrated to alter brain vascular structure and morphology (Fulop et al., 2019). Fortunately, several vascular mechanisms involved in cognitive decline are partially reversible and serve as potential therapeutic targets in aging-related cognitive disorders (Fulop et al., 2019). The structural and functional alterations of cerebral vasculature may cause ischemia, blood–brain barrier disruption, cerebral blood flow (CBF) modification, rarefaction of vasculature, increased neuroinflammation, and impaired neurovascular coupling, which, in turn, have a robust impact on the proper function of the brain (Tarantini et al., 2017; Csipo et al., 2019; Fulop et al., 2019). Notably, age-related changes in brain vasculature may derive from endothelial dysfunction (Donato et al., 2015), which are characterized by impaired endothelial-dependent dilatation (Lesniewski et al., 2009), angiogenesis (Zhuo et al., 2010), permeability (Pelegri et al., 2007), and fibrinolysis (Yamamoto et al., 2002). In addition, growing evidence recognizes the importance of oxidative stress and inflammation in endothelial functions, such as bioenergetics and mitochondrial functions (Donato et al., 2015). Finally, as mentioned above, the clearance of elevated levels of glucose and lipids is reduced in aging and is associated with acute endothelial dysfunction (Marchesi et al., 2000; Rudolph et al., 2007). It has long been known that Ca^2+^ homeostasis is crucial in the physiological functioning of cerebral microvascular endothelial cells (Stoica et al., 2021). For instance, an alteration in normal Ca^2+^ trafficking may cause mitochondrial dysfunction followed by ROS production and ATP depletion (Hong et al., 2020). In this context, TRP channels are characterized by a double role: they mediate extracellular Ca^2+^ entry and some isoforms are sensitive to ROS (Negri et al., 2019). For example, the accumulation of Aβ deposits, typically seen in AD, close to BECs may induce TRPM2-mediated intracellular Ca^2+^ signals in BECs, thereby triggering oxidative stress and modification of BBB permeability (Park et al., 2014; Mantzavinos and Alexiou, 2017). Similarly, TRP channel expression and TRP-mediated Ca^2+^ signals in BBB are modified in traumatic brain injury or stroke (Yang et al., 2019).

For all these reasons, TRP channels are important mediators of several microvascular functions and may be important tools in the prevention of aging-related cognitive deficits (Kuppusamy et al., 2021). Herein, we provide an overview of the TRP channels-dependent molecular mechanisms involved in aging and specifically in the modification of endothelial cells’ activity, highlighting their possible therapeutic role.

## 4. TRP channels in endothelial cells

### 4.1. TRP channels expression in BECs

The endothelium is an active cell layer found along all the vascular compartments. Similar to any other tissue, it can become dysfunctional, which may be caused by intrinsic and extrinsic factors (Kwan et al., 2007). Several vascular functions are regulated by endothelial cells, and specifically, by ROS-induced and Ca^2+^-dependent mechanisms (e.g., EDH, vascular permeability, and angiogenesis; Negri et al., 2021).

Transient receptor potential channels are widely express in the brain, where they are key players in the integration of several chemical and physical stimuli (Wang et al., 2020). In particular, a major fraction of TRP channels have been found in ECs (i.e., TRPC1-7; TRPV1-4; TRPA1; TRPP1-2, and TRPM1-4/6–8), but only 11 of them have been demonstrated to have a functional role in the vasculature (Thakore and Earley, 2019). Not surprisingly, TRP channel expression varies throughout the vascular tree and between different species. Furthermore, conflicting works reported that the same endothelial cell type presented different TRP expression patterns *in vitro*, supporting the hypothesis of a reorganization of ion channel and receptor expression depending on culture conditions, which is worsened by increasing passages of the cultured cells (Earley and Brayden, 2015; Negri et al., 2019). However, the TRP expression in ECs has been demonstrated *in vivo*, both in homomeric (e.g., TRPC1-6) and heteromeric (e.g., TRPC1-TRPC4; TRPV1-TRPV4; TRPC1-TRPP2-TRPV4) assembly (Negri et al., 2019).

Specifically, TRPA1 (Sullivan et al., 2015; Pires and Earley, 2018; Thakore et al., 2021), TRPM2 (Park et al., 2014), TRPP2 (Berrout et al., 2012), and TRPC3 (Kochukov et al., 2014) are expressed and have a functional role in mouse BECs. Furthermore, Luo and collaborators investigated TRPV1-4 expression in brain ECs from humans and rats and found significant differences. For instance, both human primary cultured brain microvascular cerebral ECs (BMECs) and brain microvascular endothelial cell line hCMEC/D3 (which models the human BBB, can be easily grown, and is amenable to cellular and molecular studies on cerebrovascular pathology), express more TRPV2 compared to the other isoforms. Conversely, rat cells mainly feature the TRPV4 isoform, followed by TRPV2, TRPV3, and TRPV1 in decreasing frequency (Luo et al., 2020). Herein, we focus our attention on TRP channels in BECs by highlighting their role in aging-related disorders.

### 4.2. Cerebral endothelial cell functions mediated by TRP channels

Endothelial TRP channels regulate EC functions by mediating extracellular Ca^2+^ influx and membrane potential variations in response to several chemical and physical stimuli, as reported in previous paragraphs (Sundivakkam et al., 2013). The increase in [Ca^2+^]_i_ activates multiple signaling pathways and cellular functions. For example, TRPV4 (Zhang et al., 2009), TRPP2 (Berrout et al., 2012), and TRPM2(Park et al., 2014) channels are involved in the modulation of the vascular tone, thereby mediating endothelial nitric oxide synthase (eNOS) activation and the subsequent NO release in mice BECs.

On the other hand, TRP-mediated cation influx (e.g., Na^+^ and Ca^2+^) results in membrane depolarization or in activation of K^+^ channels, inducing membrane hyperpolarization (Earley and Brayden, 2015; Smani et al., 2018; Negri et al., 2019). For instance, it has been long known that TRPC3/6 and TRPM4 have a role in vascular smooth muscular cell depolarization. Conversely, TRPV4-driven Ca^2+^ entry activates Ca^2+^-dependent K^+^ channels that cause the hyperpolarization of vascular smooth muscle cells (Earley et al., 2005; Smani et al., 2018; Negri et al., 2019). Recently, it has been demonstrated by different groups that the interplay between TRP channels and the Ca^2+^-sensitive K^+^ channels is also present in ECs, and is called the endothelium-dependent hyperpolarization (EDH) mechanism (Feletou and Vanhoutte, 2006a; Longden et al., 2017). In this context, TRPA1 induces endothelium-dependent vasodilatation and smooth muscular cell membrane hyperpolarization in mouse cerebral arteries (Earley et al., 2009), by initiating the molecular mechanism in the deepest region of the capillary network (Thakore et al., 2021). Likewise, TRPV4 (Liu et al., 2011; Zhang et al., 2013; Harraz et al., 2018), TRPC3 (Marrelli et al., 2003), and TRPV3 (Earley, 2011; Pires et al., 2015) channels interact with endothelial Ca^2+^-sensitive K^+^ channels and inward-rectifying K^+^ (K_IR_) channels, thereby causing the onset of the EDH mechanism.

In addition, TRP channels are important in vascular permeability modulation (e.g., TRPV4, TRPV1, TRPC1, TRPC4, TRPC6, and TRPM2) by regulating cell–cell adhesions and endothelial shape variations driven by the cytoskeleton recently reviewed by Genova and collaborators (Genova et al., 2020). It is particularly important if we consider the central role of the BBB in the physical brain, serving as protection from systemic inflammation (Duarte-Delgado et al., 2019). Finally, TRP channels are involved in neuroinflammation, which is closely related to vascular permeability. In this context, TRPV1, TRPV4, TRPA1, and TRPM2 show anti-inflammatory properties, while TRPC1, TRPC3, and TRPC6 have pro-inflammatory activities (Thakore and Earley, 2019).

## 5. TRP channels in cerebrovascular diseases

### 5.1. Endothelial dysfunction and oxidative stress

In paragraph 4.2, we have already mentioned the role of BEC TRP channels in vascular smooth muscle contraction/dilatation and NO availability. An alteration in this fragile equilibrium is the source of neurovascular pathologies, such as hypertension and atherosclerosis (Goligorsky, 2000; Feletou and Vanhoutte, 2006b). Notably, aging-induced cerebral endothelium dysfunction causes disorders in cerebral blood supply that is worsened by the typical increase in ROS production present in VCI (Iadecola, 2013; Balasubramanian et al., 2020). It has long been known that some TRP channels (e.g., TRPC3, TRPC4, TRPM2, TRPM7, and TRPA1) may be activated by oxidative stress (Hecquet et al., 2008; Wong and Yao, 2011). Regarding brain endothelial dysfunction, the extracellular accumulation of Aβ typically seen in AD induces oxidative stress in BECs, which activates the DNA repair enzyme poly-ADPR polymerase in cultured mouse brain endothelial cells (Park et al., 2014). The production of poly-ADPR activates TRPM2 channel, thereby inducing an endothelial Ca^2+^ overload, endothelial dysfunction, and neurovascular impairment (Park et al., 2014). Furthermore, endothelial TRPA1 channels play a crucial role in CBF regulation at the artery, arteriole, and capillary level (Kuppusamy et al., 2021). For instance, TRPA1 is activated by superoxide anions generated by the NADPH oxidase isoform 2 (NOX2) enzyme. Interestingly, TRPA1 and NOX2 colocalize only in cerebral endothelium. Moreover, endothelial TRPA1 has been shown to be activated by ROS and 4-hydroxy-2-nonenal (4-HNE), which may be produced by lipid peroxidation or released by astrocytes and neurons during neuronal activity (Earley et al., 2009; Sullivan et al., 2015). Herein, TRPA1 mediates vasodilation by activating IK and SK channels, and inwardly rectifying K^+^ (K_IR_) channels and by inducing the EDH mechanism. Thereafter, cerebral endothelial TRPA1 channels may be activated by mitochondrial ROS in mouse pial arteries and parenchymal arterioles under hypoxic conditions (Pires and Earley, 2018). These data show that TRPA1 is a valuable candidate for oxidative stress sensing, and similarly to TRPM,2 an alteration in TRPA1 functioning may be a source of Ca^2+^ overloading in BECs, causing consequent brain endothelial dysfunction.

### 5.2. TRP channels in neurovascular coupling

**Figure 1:**
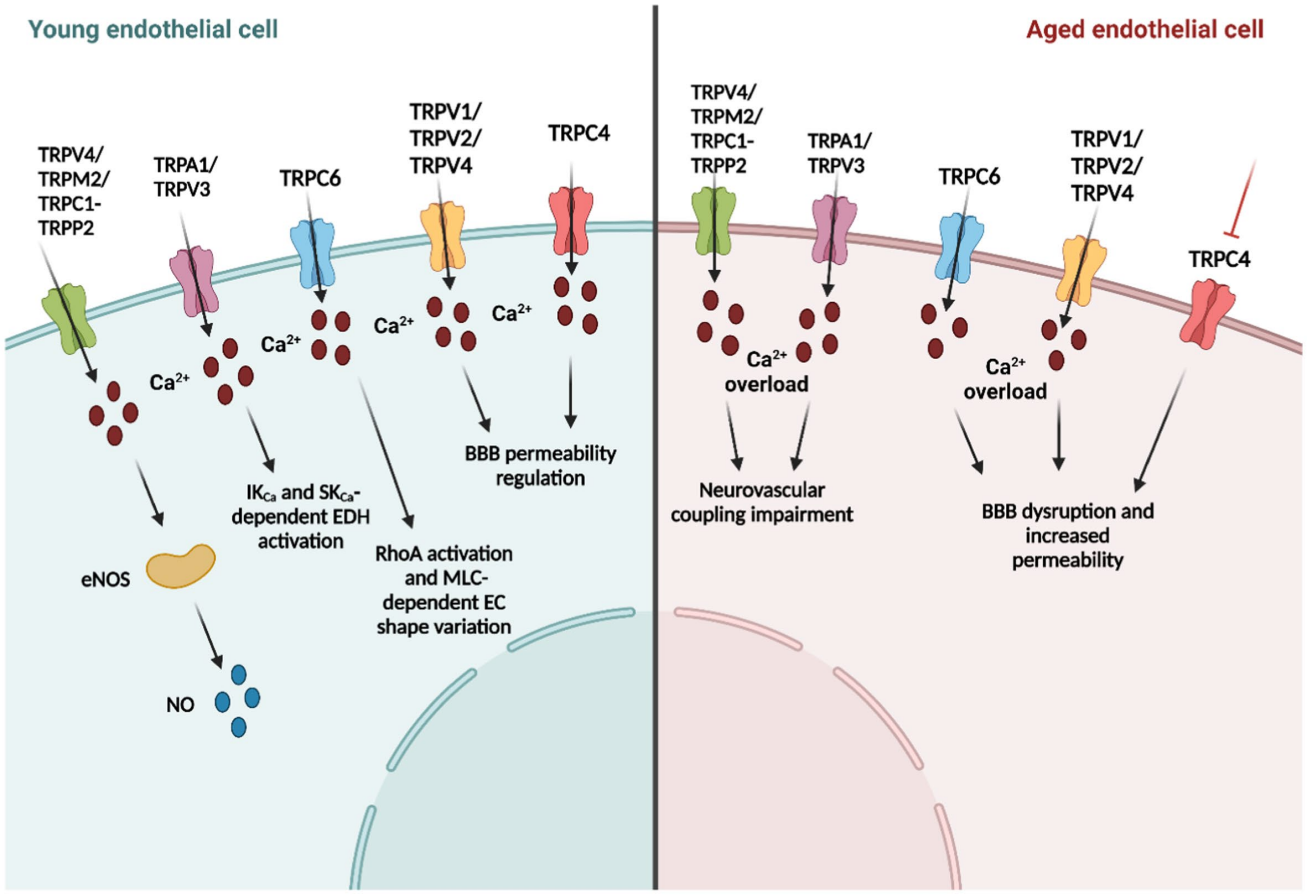
Age-related changes in endothelial transient receptor potential (TRP) channel function.

Neurovascular coupling is the mechanism whereby an increase in neuronal activity leads to an increase in CBF to ensure local supply of oxygen and nutrients to the activated areas (Iadecola, 2017). In this context, during the first Stroke Progress Review Group meeting of the National Institute of Neurological Disorders and Stroke of the NIH (2001), the concept of the neurovascular unit (NVU), or rather the functional and anatomical complex composed by ECs, basal lamina, pericytes, smooth muscular cells and neuronal cells (i.e., astrocytes, neurons, and interneurons), emerged (Iadecola, 2017; Guerra et al., 2018). NVC impairment in age-related neurodegenerative disorders has been demonstrated again and again in pre-clinical (Tarantini et al., 2021a,b) and clinical studies (Toth et al., 2022; Zhang et al., 2022) in AD and VaD (Iadecola, 2004; Iadecola, 2013). Notably, NVC is mediated by both vasoactive molecules (e.g., NO and prostacyclin) and by EDH (Iadecola, 2017), and as reported in the previous paragraphs, endothelial TRP channels have a fundamental role in these mechanisms. For instance, TRPV4 activation by arachidonic acid metabolites [e.g., 5,6 epoxyeicosatrienoic acids (5,6-EET), 8,9-EET and 11,12-EET] mediates an increase in [Ca^2+^]_i_ that activates phospholipase A2 and induces EDH-driven vasodilatation in rat middle cerebral arteries (You et al., 2002; Marrelli et al., 2007). Moreover, TRPV4-dependet Ca^2+^ signals trigger IK and SK-mediated EDH mechanism in rodent cortical arterioles in response to ATP (You et al., 2002; Liu et al., 2011) and in mouse posterior cerebral arteries in response to acetylcholine (Zhang et al., 2013). Notably, TRPV4-mediated dilation in mouse pial arteries is impaired in the mouse model of AD (Zhang et al., 2013); likewise, TRPV4 is shown to be fundamental in parenchymal arteriolar dilation and cognitive function in hypertension (Diaz-Otero et al., 2018, 2019). Furthermore, a recent investigation by Nelson’s group demonstrated that TRPV4-mediated Ca^2+^ influx, enhanced by intracellular Ca^2+^ release from the endoplasmic reticulum through InsP_3_Rs, causes a robust NO release in the arteriolar-capillary transitional zone, thereby stimulating pericyte relaxation. This mechanism results in focal dilation limited to the branch region with Ca^2+^ signals and thus in close proximity to the neuronal activity site (Longden et al., 2021). The TRPV4 channel is also expressed in capillary BECs (Longden et al., 2017; Figure 1), where it mediates Ca^2+^-dependent eNOS activation and the consequent NO release in human brain microvascular endothelial cell line (hCMEC/D3; Berra-Romani et al., 2019a). Interestingly, capillary ECs lack SK and IK channels (Longden et al., 2017), and indeed, TRPV4 causes endothelial depolarization by conducting a Ca^2+^ influx. However, recently, endothelial K_IR_2.1 channels have emerged as important players in CBF regulation since, unlike IK and SK channels, they are expressed in capillary BECs. For instance, Harraz et al. have reported that Gq protein-coupled receptor (GqPCR)-mediated phosphatidylinositol 4,5-bisphosphate (PIP2) hydrolysis activated TRPV4 channels and simultaneously inhibited K_IR_2.1 channel activity in mouse capillary BECs (Harraz et al., 2018). Several GqPCR agonists (e.g., acetylcholine, ATP, and PGE2) are released by astrocytes and neurons during NA (Harraz et al., 2018), and they may be fundamental in endothelial membrane repolarization after the onset of the hyperpolarizing signal. Indeed, they act by inhibiting K_IR_ channels, and consequently, reduce the retrograde propagation of the electric signal by activating TRPV4-dependent endothelial depolarization (Harraz et al., 2018).

Another important TRP channel involved in NVC is TRPA1 channel, expressed in rat cerebral arteries-MEGJs, where it co-localizes with IK and SK channels and participates to the onset of the EDH phenomenon (Sullivan et al., 2015). For instance, the dietary compound allyl isothiocyanate (AITC) selectively activates TRPA1, and the consequent IK opening that induces vasodilation in rat cerebral arteries (Earley et al., 2009; Qian et al., 2013). Furthermore, endothelial TRPA1 is responsible for neuronal activity sensing and NVC initiation in deep brain capillaries through a ROS-dependent mechanism (Thakore et al., 2021). Herein, 4-HNE-induced TRPA1 activation elicits an increase in [Ca^2+^]_i_, which is propagated to the upstream arterioles through the Ca^2+^-dependent ATP release *via* pannexin 1 (Panx1; Thakore et al., 2021). ATP, in turn, binds to P2X receptors in adjacent cells, thereby spreading the Ca^2+^ wave until it reaches the arterioles. Herein, the chemical signal is converted into the hyperpolarizing electric signal by IK and SK channels (Pires and Earley, 2018; Alvarado et al., 2021). Likewise, EDH generation may be induced by TRPC3 and TRPV3 in cerebral arteries (Earley and Brayden, 2015). TRPC3 is stimulated by the phospholipase C product Diacylglycerol (DAG; Negri et al., 2019), and is responsible for the ATP-induced hyperpolarization and consequent vasodilatation in mouse middle cerebral arteries and posterior cerebral arteries through a TRPA1-like mechanism. Specifically, IK channels drive the initial phase of endothelial hyperpolarization, and conversely, SK channels sustain the following delayed hyperpolarization phase (Kochukov et al., 2014). Likewise, TRPV3 elicits IK and SK channels opening and triggers EDH in rat isolated posterior cerebral and superior cerebellar arteries, but, unlike TRPA1, it is mainly localized on the endothelial membrane and not in MEGJs (Earley, 2011; Pires et al., 2015). Of note, TRPV3-dependent Ca^2+^ signals were significantly higher compared to other TRP channels (e.g., three times bigger than TRPV4-induced and 1.5 times bigger than TRPA1-induced Ca^2+^ sparklets). Finally, Berrout and collaborators showed that stretch-induced Ca^2+^ signals and NO production in mouse BMECs were significantly reduced by TRPC1 and TRPP2 pharmacological and genetic manipulation (Berrout et al., 2012), suggesting an additional activation mechanism of TRP channels involved in endothelial NO-dependent vasodilation.

### 5.3. BBB disruption and neuroinflammation

The BBB is a specialized vasculature that separates the brain from peripheral circulation. The BBB presents a limited permeability to macromolecules and peripheral immune cells by exerting a protective effect against neuroinflammation (Chen et al., 2020). The NVU is responsible for the formation and maintenance of the BBB^10^, of which malfunction and disruption have been shown to be involved in age-related neurodegeneration(Iadecola, 2013; Kerkhofs et al., 2021; Towner et al., 2021; Montagne et al., 2022) in several neuropsychiatric, vascular, metabolic, and immunologic diseases(Luo et al., 2020). Notably, BBB integrity is regulated by BECs calcium signals, which may be largely mediated by TRP channels (Negri et al., 2019). For instance, TRPV channels, and especially TRPV2 in humans and TRPV4 in rodents, have been demonstrated to have a major role in BBB integrity (Luo et al., 2020). Indeed, the potent TRPV2 antagonist cannabidiol (CBD) prevents the BBB disruption induced by oxygen glucose deprivation (OGD) in a human *in vitro* model of BBB, consisting of human BMECs and human astrocytes coculture (Hind et al., 2016). A subsequent work showed that CBD-induced TRPV2 activation modulates human BBB permeability defined by transendothelial electrical resistance (TEER; Luo et al., 2019). On the other hand, TRPV4 influences mouse BBB permeability by sensing the osmotic changes and BMECs volume alteration (Brown et al., 2008). In this context, TRPV4 activation may assume a role in driving Ca^2+^ entry in ECs and regulating BBB functions (Cig et al., 2021). For instance, Friese’s group has shown an amelioration of BBB transendothelial resistance after TRPV4 pharmacological inhibition in mouse BMECs (Rosenkranz et al., 2020). This effect is abrogated by the TRPV4 downregulation with interferon-γ and tumor necrosis factor-α. Moreover, *in vivo* treatment did not prevent BBB dysfunction in a TRPV4 knock-out mouse model of multiple sclerosis or in MCAO/R-induced brain damage (Rosenkranz et al., 2020). Accordingly, Jie and collaborators have demonstrated that the injection of the potent TRPV4 agonist, GSK1016790A, caused BBB disruption in mice. Notably, the effect of GSK1016790 is prevented by co-injecting the TRPV4 antagonist HC-067047(Jie et al., 2015). Taken together, these data demonstrate that TRPV4 regulates BBB integrity upon different non-physiological conditions (Rosenkranz et al., 2020). Regarding TRPV1, it has been demonstrated to be scarcely expressed in BECs; however, the co-culture of human primary BMECs with primary astrocytes from the same patient increased TRPV1 expression by 4.8-fold (Luo et al., 2020), which confirms the importance of the cellular interplay within the NVU. Early work from Zygmunt and collaborators have shown that anandamide-dependent TRPV1 activation reduced human BBB permeability, thereby stimulating the release of the vasodilator neuropeptide, calcitonin gene-related peptide (CGRP; Zygmunt et al., 1999; Hind et al., 2016). However, subsequent investigations have demonstrated the opposite role of TRPV1 in BBB permeability regulation (Hu et al., 2005; Yang et al., 2019). An earlier work has shown that the potent TRPV1 agonist, capsaicin, induced an increase in BBB permeability in an *ex vivo* rat model (Hu et al., 2005). Therefore, recently, Yang and collaborators have provided the evidence that TRPV1 inhibition with the specific blocker capsazepine, avoids BBB disruption in an *in vivo* mouse model of TBI (Yang et al., 2019).

Transient receptor potential conical channels have also been correlated to BBB function maintenance (Li and Ehrich, 2013; Ryu et al., 2013; Kuppusamy et al., 2021). Multiple studies have put forward TRPC6 as a regulator of endothelial permeability. For instance, DAG-induced TRPC6-mediated Ca^2+^ entry stimulates the Ras homolog family member A (RhoA), the following myosin light chain-dependent EC shape variation, and the raising gap formation between adjacent cells (Singh et al., 2007; Figure 1). Furthermore, the TRPC3 channel is overexpressed in the rat brain’s piriform cortex ECs in status epilepticus. TRPC3 channel upregulation reduces the expression of SMI-71, a rat endothelial BBB antigen correlated with BBB disruption and neuronal damage (Ryu et al., 2013). Conversely, the insecticide Chlorpyrifos reduces TRPC4 channel expression by inducing BBB disruption in Sprague–Dawley rats (Li and Ehrich, 2013). Moreover, there is an emerging theory regarding the protein interaction between TRP channels and scaffold proteins. In this view, TRP channels may interact with caveolin-1, which is overexpressed after an ischemic insult, thereby leading a major BBB permeability. Caveolin-1 is an important structural protein of the endothelial caveolae, which are essential elements in neurovascular coupling (Chow et al., 2020). It has been shown to anchor eNOS, which, in turn, is regulated by TRP channels activation (Goligorsky et al., 2002; Daneva et al., 2021). However, further investigations are needed to understand the molecular interaction and interdependence of these three proteins in the context of neurovascular coupling and cerebral blood flow regulation. In summary, these data demonstrate that TRP channels are important modulators of BBB permeability.

### 5.4. TRP channels in ischemic brain damage

Elderly individuals are particularly at risk for stroke; in the United States alone, it is estimated that strokes kill one person every 4 min (Shekhar et al., 2021; Kaucsar et al., 2022). Recently, Zhu’s group reviewed the role of TRPC channel during ischemic episodes (Jeon et al., 2020). The major studies are conducted on middle cerebral artery occlusion/reperfusion (MCAO/R) in an *in vivo* rat model of ischemic stroke with middle carotid artery occlusion followed by reperfusion. Likewise, multiple groups have performed oxygen glucose deprivation/re-oxygenation (OGD/R) assays on *in vitro* models of ischemia/reperfusion, which mimic neuronal death induced by oxygen and glucose deprivation followed by re-oxygenation. These investigations are then combined with additional *in vivo* evaluations of TRPC knock-out mice or with the *in vitro* pharmacological and genetic manipulation of TRP channels. Of note, TRPC3, TRPC4, TRPC6, and TRPC7 have been shown to play a key role in ischemic brain damage (Jeon et al., 2020). Although, a controversial result from Xu et al. have demonstrated that genetic deletion of TRPC1 ameliorates OGD/R-induced neuronal death (Xu et al., 2018), it seems that TRPC1 has a protective role in ischemic damage by reducing the production of ROS. Conversely, ROS-dependent TRPA1 activation results in the limitation of ischemic brain damage (Pires and Earley, 2018; Alvarado et al., 2021). For instance, hypoxia promotes mitochondrial ROS production followed by lipid peroxidation and TRPA1-mediated vasodilation in cerebral pial arteries and intraparenchymal arterioles (Pires and Earley, 2018). This could result from the angiogenic function of TRPC5, which dampens injury-induced inflammation. Indeed, TRPC5 has been reported to promote endothelial cell sprouting, angiogenesis, and blood perfusion in ischemic tissues through activation of nuclear factor of activated T cell (NFAT) isoform c3 and angiopoietin 1(Zhu et al., 2019; Figure 1).

Transient receptor potential vanilloid channels may also interfere with ischemic episodes through a controversial TRPV1 role in this process. On the one hand, pre-ischemia treatment with capsaicin, a selective TRPV1 channel agonist, protects the Mongolian gerbil’s brain from global ischemia (Pegorini et al., 2005). Accordingly, Khatibi and collaborators have confirmed the neuroprotective effect of TRPV1 channel in another rat model of brain ischemia. Herein, capsaicin pre-treatment reduced the infarcted area and normalized vessel reactivity (Khatibi et al., 2011). On the other hand, more recent investigation demonstrates that post-ischemia TRPV1 inhibition limited neuronal damage by decreasing toll-like receptor 2 (TLR2) and TLR4 (Hakimizadeh et al., 2017), which are usually upregulated after brain ischemia and modulate inflammation and neuronal death (Lehnardt et al., 2007). Accordingly, TRPV1 inhibition has been demonstrated to have a neuroprotective role during brain ischemia in mice, and its expression is enhanced post-stroke (Luo et al., 2020). In addition, CBD elicited a protective effect in human BMECs upon OGD, but the investigation did not demonstrate the direct activation of TRPV2 in BMECs (Hind et al., 2016). Likewise, the pharmacological inhibition of TRPV4 with HC-067047 ameliorates BBB disruption in rats with focal cerebral ischemia and reperfusion (Xie and Lu, 2018). TRPV4 inhibition or genetic deletion is also responsible for reduced BBB disruption after intracerebral hemorrhage in rats, which is reputed to be a fatal stroke subtype (Zhao et al., 2018). Finally, TRPM4 is found to be upregulated in vascular endothelium in the penumbra region of a rat model with permanent middle cerebral artery occlusion (Loh et al., 2014). In addition, the genetic silencing of TRPM4 increases angiogenesis and capillary integrity in the same animal model (Loh et al., 2014). Based on the reported data, further work is needed to clarify some roles of TRP channels (e.g., TRPC1 and TRPV1) in ischemic episodes. Nevertheless, they are likely to be promising therapeutic targets to treat stroke-induced brain damage.

## 6. TRP channels as promising targets in the treatment in age-related cerebrovascular disorders

Previous paragraphs underlined the important role of endothelial TRP channels in the onset of microvasculature defects linked to cognitive decline (summarized in Table 1). As already mentioned, with these conditions, the pathological environment is characterized by the presence of higher levels of ROS and dysregulated Ca^2+^ homeostasis (Hong et al., 2020). The major studies done to understand the involvement of TRP channels in neurodegenerative disorders have been done on TRP-KO mice and by using TRP channels agonists and antagonists. In this view, we can delineate the role of TRP channels in several neurological diseases, but we cannot rule out their specific involvement in the malfunction of different cell types that are impaired in the disorders (Hong et al., 2020; Koivisto et al., 2022). For instance, Jang et al. have demonstrated that genetic elimination and pharmacological inhibition of TRPA1 in mice brain had a neuroprotective role on stroke-derived hypoxia and reduced myelin damage (Hamilton et al., 2016). On the contrary, TRPV1-dependent hypothermia has been shown to reduce stroke volume by 50% and increase the post-ischemia recovery in mice (Cao et al., 2017). Regarding cognitive decline, Borbély and collaborators have revealed that aging Trpa1^−/−^ mice exhibited an improved memory (Borbely et al., 2019). In addition, TRPA1 antagonism may be a potential therapeutic target for AD-associated seizures (Payrits et al., 2020). Similarly, triple-transgenic AD mouse model KO for Trpv1 showed an ameliorated memory function (Kim et al., 2020). Finally, a recent work by Thapak and collaborators showed that Tranilast, a powerful TRPV2 inhibitor, ameliorates cognitive impairment in a rat model of AD, delineating TRPV2 as a potential therapeutic target for AD^137^.

**Table 1:** Endothelial transient receptor potential (TRP) channels involved in cerebrovascular functions.

Channel	Stimulation/manipulation	Localization	Microvascular mechanism	Pathological condition	Ref
TRPM2	poly-ADPR	Mice BECs	Ca^2+^ overload, endothelial dysfunction	Alzheimer’s disease and neurovascular coupling impairment	Park et al. (2014)
TRPM4	Genetic silencing	Rat middle cerebral artery	Increased angiogenesis and capillary integrity	Stroke	Loh et al. (2014)
TRPA1	4-HNE, mitochondrial ROS and AITC	Mouse and rat cerebral pial arteries and parenchymal arterioles	IK, SK and K_IR_ channels activation and vasodilation through EDH mechanism	Lipid peroxidation and hypoxic condition	Qian et al. (2013); Pires and Earley (2018)
	4-HNE and AITC	Mouse capillary BECs	Neurovascular activity sensing and upstream propagation of the Ca^2+^ wave	Neurovascular coupling impairment	Thakore et al. (2021)
	Mitochondrial ROS and 4-HNE	Mouse cerebral pial arteries and intraparenchymal arterioles	N.I.	Ischemic brain damage limitation	Sullivan et al. (2015); Pires and Earley (2018)
TRPV4	Arachidonic acid metabolites, ATP and acetylcholine,	Mouse middle cerebral arteries and mouse posterior cerebral arteries	Ca^2+^ overload, PLA2 activation and vasodilatation through EDH mechanism	Alzheimer’s disease and hypertension	Zhang et al. (2013); Diaz-Otero et al. (2018); Diaz-Otero et al. (2019)
	PIP2 hydrolysis	Mouse capillary BECs	K_IR_2.1 inhibition and endothelial cell depolarization and eNOS-dependent NO release	Neurovascular coupling impairment	Harraz et al. (2018); Longden et al. (2021)
	GSK1016790	Mouse BMECs	Ca^2+^ overload	Multiple sclerosis, brain edema, and BBB dysfunction	Jie et al. (2015); Rosenkranz et al. (2020)
	Inhibition with HC-067047	*In vivo* rat model of intracerebral hemorrhage	N.I.	Amelioration in BBB disruption after intracerebral hemorrhage	Zhao et al. (2018)
TRPV3	Oregano	Rat cerebral and superior cerebellar arteries MEGJs	IK and SK channels activation and EDH mechanism (neurovascular coupling)	Neurovascular coupling impairment	Earley (2011); Pires et al. (2015)
TRPV2	Inhibition with CBD	*In vitro* model of BBB and human BMECs	Modulation of BBB permeability	BBB disruption	Hind et al. (2016); Luo et al. (2019)
	Inhibition with Tranilast	Rat model of Alzheimer’s disease	N.I.	Amelioration	Thapak et al. (2022)
				Alzheimer’s disease-dependent cognitive impairment	
TRPV1	Anandamide	Rat primary BMECs	Release of the vasodilation neuropeptide CGRP	Reduction of BBB permeability	Zygmunt et al. (1999)
	Capsaicin	*Ex vivo* rat model	N.I.	Increase in BBB permeability	Hu et al. (2005)
	Capsazepine-dependent inhibition	*In vivo* mouse model of TBI	N.I.	No BBB disruption	Yang et al. (2019)
	Pre-ischemia capsaicin treatment	*In vivo* Mongolian gerbil and rat brain	N.I.	Global ischemia prevention and reduction in the infarcted area	Pegorini et al. (2005); Khatibi et al. (2011)
	Post-ischemia TRPV1 inhibition	*In vivo* rat middle cerebral artery	Decrease in TLR2 and TLR4 receptors expression	Limitation of neuronal damage	Hakimizadeh et al. (2017); Luo et al. (2020)
TRPC1-TRPP2	Pharmacologic and genetic manipulation	Mouse bEnd3	Ca^2+^ overload and NO production	Neurovascular coupling impairment	Berrout et al. (2012)
TRPC1	Genetic deletion	Mice Trpc1^−/−^	Reduction of ROS production	Protective role in ischemic damage	Xu et al. (2018)
TRPC3	DAG	Mouse middle cerebral arteries and posterior cerebral arteries	ATP-induced hyperpolarization and vasodilatation	Neurovascular coupling impairment	Kochukov et al. (2014)
TRPC4	Channel overexpression	Rat piriform BECs	Reduction of SMI-71 expression	Reduction of BBB disruption	Ryu et al. (2013)
	Chlorpyrifos-dependent reduction of channel expression	Rat BECs (RBE4)	Reduction of TRPC4, claudin5 and ZO1 expression	BBB disruption	Li and Ehrich (2013)
TRPC6	DAG	Human ECs	Ca^2+^-dependent RhoA activation and myosin light chain-mediated EC shape variation	Increase in BBB permeability	Singh et al. (2007)

Schematic summary of the main transient receptor potential (TRP) channels functions in the cerebral vasculature. BECs, brain endothelial cells; BMEC, brain microvascular endothelial cells; AITC, allyl isothiocyanate; EDH, endothelium-derived hyperpolarization; I(S)K, intermediate (small) conductance Ca^2+^-activated potassium channels; K_IR_, inward-rectifier potassium channels; CGRP, calcitonin gene-related peptide; PLA2, phospholipase A2; PIP2, phosphatidylinositol 4,5-bisphosphate; eNOS, endothelial nitric oxide synthase; MEGJs, myoendothelial gap junctions; N.I., not investigated; BBB, blood–brain function; CBD, cannabidiol; TBI, traumatic brain injury; TLR, toll-like receptors; DAG, diacylglycerol.

In this review, we have clearly shown the importance of TRP channels in microvasculature mechanisms. Nevertheless, there are not specific therapies targeting endothelial TRP channels currently. As mentioned above, the main problem is to selectively silence or activate TRP channels on endothelial cells without affecting other cell types. An elegant example of TRP stimulation characterized by temporal and spatial precision is represented by the optical stimulation of endothelial colony forming cells (ECFCs) plated on a light-sensitive organic semiconductor (poly(3-hexylthiophene-2,5-diyl), P3HT)(Lodola et al., 2019; Negri et al., 2022). Intriguingly, the authors have demonstrated that the optical stimulation of P3HT thin films can induce the Ca^2+^-dependent increase of ECFCs proliferation and tubulogenesis *in vitro* through the ROS-dependent TRPV1 activation (Lodola et al., 2019; Negri et al., 2022). In addition, the two investigations have shown that the role of ROS in the phototransduction mechanism is important if we consider the fact that several ROS mechanisms(?) may be activated by nontoxic ROS levels (Negri et al., 2019). Notably, organic semiconductors thin films may be used as implantable patches to stimulate the region of interest, or conversely, they may be engineered as nanoparticles and function to selectively target endothelial cells, avoiding the previously mentioned problem of non-specificity.

Finally, several works highlight the importance of different nutrition regimens (e.g., caloric restriction and time restricted feeding) in the improvement of aging-related neurovascular disorders (Csiszar et al., 2014; Balasubramanian et al., 2020; Oikawa et al., 2021; Dobreva et al., 2022; Maroto-Rodriguez et al., 2022). In this context, TRP channels are intriguing molecular elements, since they may be activated by several dietary compounds (Negri et al., 2019). Several food-derived molecules stimulate TRPV channels, such as: (i) capsaicin and piperine from pepper(Kobata et al., 1999), eugenol from cloves(Yang et al., 2003) and gingerol from ginger(Kim et al., 2016) activate TRPV1; (ii) eugenol and carvacrol from oregano stimulate TRPV3(Xu et al., 2006); and (iii) apigenin (Ma et al., 2012) and bisandrographolide A target TRPV4(Akbar, 2011). Apigenin is a plant-derived flavone that stimulates endothelial TRPV4 to ameliorate hypertension (Ma et al., 2012). Additionally, TRPV4 activity is modulated by dietary ω-3 polyunsaturated fatty acids intake, ω-3 eicosanoid epoxide derivatives are required for TRPV4 function in worm neurons, and eicosapentaenoic acid enhances TRPV4 activity in human endothelial cells (Caires et al., 2017). Besides TRPV channels, TRPA1 may also be bound by different reactive electrophiles, including allyl isothiocyanate that is present in mustard oil and pungent vegetables (e.g., cauliflower, radish, wasabi, and cabbage; Appendino et al., 2008); aldehyde cinnamaldehyde from cinnamon (Bandell et al., 2004); and polygodial that is obtained from different varieties of pepper (Escalera et al., 2008). Finally, also TRPC channels activated by DAG (e.g., TRPC3, TRPC6, and TRPC7) may be influenced by a lipid diet, which introduces many edible fats and lipids (e.g., Olive oil and nuts) rich in DAG (Trebak et al., 2007). Although there are several pieces of evidence about TRP channels and food-derived molecules, the field remains controversial, requiring a lot more work to understand the effective role of these relationships in several cardio- and neurovascular disease.

## 7. Conclusion

Recent evidence in both animal models and humans, which has been highlighted in this review, suggest that TRPs in the brain cerebrovasculature are one of the key cellular mechanisms that mediate age-related endothelial dysfunction, leading to poor cognitive outcomes in elderly individuals. Endothelial TRP channels are not only key players in physiological and pathological vascular functions thanks to their ability to sense a wide spectrum of chemical and physical stimuli (Negri et al., 2019), but also are emerging as fundamental regulators of endothelial-dependent dilation, vascular permeability, and neuroinflammation. This is particularly interesting in age-related microvascular disorders, such as VCI, and related neurodegenerative diseases, such as dementia, which are characterized by alterations in cerebral vascular functions (Balasubramanian et al., 2020). There is evidence that TRP channels in the cerebrovascular system can be modulated by both endogenous and exogenous agents. This suggests that targeting TRP channel activity through dietary and pharmacological interventions may be a viable approach for restoring cerebrovascular function. Lifestyle interventions, such as dietary regimens, dietary compounds, and exercise, have been shown to improve health span, and could be beneficial for targeting TRP channels in the treatment of age-related VCI and related dementias. Specifically, endothelial TRP channels may be promising targets for dietary and pharmacological studies. Further research is needed to investigate the effects of dietary compounds, such as oregano, pepper, and green tea, on brain endothelial TRP channels.

## Author contributions

SN, MS, and ST contributed substantially to the conception or design of the manuscript. SN, MS, and ST contributed to the generating, drafting, and revising the intellectual content and provided approval for publication of the content. HS contibuted in generating, drafting, and revising the intellectual content and provided approval for publication of the content All authors contributed to the article and approved the submitted version.

## Funding

This work was supported by grants from the National Institute on Aging (NIA R03AG070479 and NIA K01AG073614 to ST), the American Heart Association AHA CDA941290 to ST, the NIA-supported Geroscience Training Program in Oklahoma (T32AG052363), the NIA-supported Oklahoma Nathan Shock Center, the NIGMS supported Center of Biomedical Research Excellence (CoBRE) (1P20GM125528-01A1), and the NCI Cancer Center Support Grant (P30 CA225520) and the Oklahoma Tobacco Settlement Endowment Trust.

## Conflict of interest

The authors declare that the research was conducted in the absence of any commercial or financial relationships that could be construed as a potential conflict of interest.

## Publisher’s note

All claims expressed in this article are solely those of the authors and do not necessarily represent those of their affiliated organizations, or those of the publisher, the editors and the reviewers. Any product that may be evaluated in this article, or claim that may be made by its manufacturer, is not guaranteed or endorsed by the publisher.
